# Roof Gardens

**Published:** 1901-11-23

**Authors:** 


### ROOF GARDENS.

In The Hospital for August 3rd an annotation appeared under the heading "Roof Conservatories on Town Houses," pointing out the possibility of utilising what would under ordinary circumstances be waste spaces. On the 31st of the same month we inserted a letter from Messrs. Frankenberg and Co., of 37 Great Dover Street, S.E., manufacturers of "Inodorite," which stated that the firm laid roofs suitable for gardens or play-grounds, and offering through the medium of the paper to lay such a roof, not exceeding 100 square yards, on any hospital in London that cared to avail itself of the offer. This promise has now been fulfilled, and the new building of the Hospital for Epilepsy and Paralysis, which is being erected in Maida Vale, is the recipient of the gift. We have inspected the roof, which is laid over the out-patients' department, and presents at present the appearance of a gravelled square surrounded by a stone coping, on which, we are informed, a railing will eventually be placed. The roof, which opens off one of the wards, will be used as an airing ground for the patients. At the unfinished edge we saw a section of the floor, which had a concrete layer below the "Mastic Sheeting"; the roof being supported on steel girders.

The manufacturers claim for their system certain advantages. They state that, owing to the material being elastic, atmospheric action upon the steel, wood, or iron joists does not disturb it; that the materials used are non-conductors, so that the "temperature is much cooler in summer and warmer in winter" than is the case when other floors are used; that the mastic sheeting covering, laid by the Mastic Astrictum Process, can be applied to any surface on existing buildings without any preparations; that it will last as long as an ordinary building; that, owing to the slight fall required, viz., 1 in 37, it offers no resistance to the wind; that flowers and even shrubs may be grown on it; and, finally, that it costs about half as much as asphalt. Time must be allowed for a fair test; and in the meanwhile we can only say that we shall hope, after a further visit to the hospital in question, to endorse all that the manufacturers claim for the invention.

